# Serum Calreticulin Is a Negative Biomarker in Patients with Alzheimer’s Disease

**DOI:** 10.3390/ijms151221740

**Published:** 2014-11-25

**Authors:** Qiao Lin, Yunpeng Cao, Jie Gao

**Affiliations:** 1 Department of Internal Medicine, the Fourth Affiliated Hospital of China Medical University, Shenyang 110005, China; 2 Neural Department of Internal Medicine, the First Affiliated Hospital of China Medical University, Shenyang 110001, China; E-Mail: caoyun2@163.com; 3 Department of Anatomy, the First Affiliated Hospital of China Medical University, Shenyang 110001, China; E-Mail: gaojie3@163.com

**Keywords:** Alzheimer’s disease, serum calreticulin, negative biomarker, real-time quantitative reverse transcriptase-PCR, ELISA, western blot

## Abstract

Calreticulin is down-regulated in the cortical neurons of patients with Alzheimer’s disease (AD) and may be a potential biomarker for the diagnosis of AD. A total of 128 AD patients were randomly recruited from May 2012 to July 2013. The mRNA levels of calreticulin were measured from the serum of tested subjects using real-time quantitative reverse transcriptase-PCR (real-time qRT-PCR). Serum levels of calreticulin were determined by ELISA and Western Blot. Serum levels of calreticulin in AD patients were significantly lower than those from a healthy group (*p* < 0.01). The baseline characters indicated that sample size, gender, mean age, diabetes and BMI (body mass index) were not major sources of heterogeneity. The serum levels of mRNA and protein of calreticulin were lower in AD patients than those from a healthy group, and negatively associated with the progression of AD according to CDR scores (*p* < 0.01). Thus, there is a trend toward decreased serum levels of calreticulin in the patients with progression of AD. Serum levels of calreticulin can be a negative biomarker for the diagnosis of AD patients.

## 1. Introduction

The global prevalence of Alzheimer’s disease (AD) has been estimated to be over 24 million, and the number is predicted to be double within two decades [1]. As the population continues to age in the world, the number of people with AD is still increasing, particularly among the elderly population. AD patients typically have impaired declarative memory [2]. One of the hallmarks of AD is that amyloid plaques in brain are usually surrounded by neurofibrillary tangles [3]. The etiology of AD remains unknown, but it is more likely to be caused by both genetic and environmental elements. For instance, there is a strong genetic basis for late-onset AD and APOE4 allele has been identified to be associated with risk of AD [4,5]. Another example, neurons live in an environment of neuronal degeneration in AD patients, which can be partially caused by diverse environmental stressors, such as psychological stress [6], anesthesia [7] and glucose hypometabolism so on [8]. All these stressors can promote tau hyperphosphorylation, amyloid-beta (Abeta) aggregation and oligomerization, which is tightly associated with the pathogenesis of AD [9].

To define best treatment strategies, early pathological diagnosis is very important for correct and on-time treatment of AD. The neuropathological effects of AD can be observed using autopsy, but confirming diagnostic and prognostic biomarkers in AD patients is not straightforward [10]. The cerebrospinal fluid (CSF) biomarkers total tau, phosphorylated tau, and 42 amino acid form of Abeta (Abeta42), have enough accuracy for the early diagnosis of AD [11]. Current AD guidelines have recommended that criteria for the diagnosis of AD should be that the levels of increased phosphorylated tau and total tau, and reduced Abeta42 are measured in CSF [12]. However, a spinal tap is required to get CSF [13]. Furthermore, obtaining biopsies is often invasive and more difficult to conduct. Not all patients like to accept the diagnosis, so researchers are still exploring other effective methods.

Presently, some potential biomarkers for AD are widely reported. For instance, DNA damage is related with the progression of AD. Biomarkers for DNA damage (chitinase and stathmin) are significantly increased in AD patients compared to healthy subjects [14]. Comparatively, a serum biomarker can be measured using a non-invasive method and more patients like to accept it. Therefore, many serum biomarkers for the diagnosis of AD patients have been exploited such as insulin-like growth factor I (IGF-I). IGF-I can enter mammalian brains and promote clearance of amyloid peptides known to accumulate in the brains of AD patients. Low levels of IGF-I have been observed in AD patients compared to healthy subjects. IGF-I can be developed as a potential biomarker in AD patients [15]. In a further example, one serum protein, activity-dependent neuro-protector homeobox protein (ADNP) is reduced in AD patients compared to healthy subjects [16]. ADNP can maintain cell survival via the modulation of p53 levels [17]. It also protects cerebral cortical neurons against Abeta42, and inhibits Abeta42 aggregation in the brain of AD patients [17]. The serum levels of protein are a direct indicator of AD. ADNP plays a critical role in preventing the progression of AD. The recent progresses in the use of AD biomarkers, which provide strong evidence for the disease, have stimulated the novel research criteria that re-conceptualizes the diagnosis involving both aspects: cognitive changes and structural/biological evidence of AD pathogenesis [18].

Further research of the biology of AD and improved diagnostic techniques are still critical to develop reliable biomarkers for the diagnosis of AD. Calreticulin is endoplasmic reticulum (ER) resident protein 60, which is encoded by CALR gene. Calreticulin can binds Ca^2+^ (a second messenger during signal transduction) and render it inactive. Calreticulin also binds misfolded proteins and prevents them from being transferred from ER to Golgi apparatus. More importantly, calreticulin regulates gene transcription via nuclear hormone receptors [19]. Therefore, calreticulin is a multifunctional protein, which involves many physiological activities of cells. A previous report finds that calreticulin is an important protein in human brain, and low levels of calreticulin are observed in the brains of AD patients, suggesting the down-regulation of calreticulin will lead to the pathological processes of AD [20]. Furthermore, calreticulin is also a cell surface scavenger receptor and can interact with Abeta [21], which is the therapeutic target of AD [22]. All the information suggests that calreticulin is an important biomarker for preventing the development of AD. Here, we explore the possibility for using serum calreticulin as a biomarker to predict the occurrence of AD. This biomarker may contribute to the early diagnosis of AD.

## 2. Results and Discussion

### 2.1. Demographic Characters

Table 1 showed demographic characters of AD patients and healthy subjects. A total of 128 AD patients received medical history and neurological examination (male/female 58/70; mean age of 66.4 years, range 45.4–87.4) (Table 1). All 130 controls were cognitively intact, after receiving medical history and neurological examination (male/female 60/70; mean age of 65.1 years, range 44.1–86.1).

Controls matched AD cases well if considering the effects of sex and age (*p* > 0.05). Previous work finds that BMI is related with the development of AD [23,24]. To avoid the heterogeneous influence of BMI on our results, the healthy subjects were selected to make sure that there were no significant statistically differences between AD patients and healthy participants (*p* > 0.05) (Table 1). Thus, BMI was not a risk factor of AD patients. Diabetes affects many elderly people in the world, with a great decrease in life quality. Diabetes has been found to affect a brain and to be a contributing risk for the development of AD [25,26]. In the same case, Blood pressure and heart disease have also been reported as risk factors of AD [27,28]. Furthermore, smoking is associated with a decrease of gray matter density in brain regions, which contributes to the incipient AD [29]. Drinking alcohol also can impair the brain function of individuals and cause dementia and geriatric cognitive disease [30,31]. Individuals with longer education have better cognitive function than those with less education in AD patients [32,33]. Compared to right handers, left handers reduce the risk of AD [34]. During the recruiting period, all these factors were also carefully considered to avoid heterogeneous effects on final results (Table 1).

Neuropsychological tests indicated that all AD subjects had MMSE scores <24 while all healthy participants had MMSE scores of >27. All AD participants had an overall CDR scale of 0.5 or over 0.5, and none was zero while overall CDR scale of all healthy subjects was zero (Table 1). These results implied that all AD patients had clinical cognitive disorders while all healthy subjects were free of clinical cognitive disorders.

**Table 1:** Baseline characters of study patients and healthy subjects.

	AD Patients (*n* = 128)	Healthy Subjects (*n* = 130)	Statistical Value	*p*-Value
Gender (male/female)	58/70	60/70	7.3 ^a^	0.825
Age (years)	65.4 ± 21.0	65.1 ± 21.0	0.21 ^b^	0.933
BMI (kg/m^2^)	24.9 ± 4.8	25.6 ± 5.1	0.34 ^b^	0.900
Diabetes/Non-diabetes	12/116	12/118	91.9 ^a^	0.898
High blood pressure/Normal blood pressure	28/100	30/100	84.0 ^a^	0.825
Coronary heart disease/Non-coronary heart disease	26/102	27/103	89.8 ^a^	0.875
Smoker/Non-smoker	48/80	50/80	82.8 ^a^	0.923
Drinker/Non-drinker	40/88	43/87	64.9 ^a^	0.881
Educational years	11.6 ± 3.2	10.9 ± 4.1	1.2 ^b^	0.266
Left-handed/Right handed	12/116	14/116	119.0 ^a^	0.872
During of illness (years)	9.9 ± 6.6	-	-	-
Pathological MRI (%)	77 (60%)	-	-	-
Neuropsychological impairment (2 T-scores 35) (%)	90 (70%)	-	-	-
MMSE scores	19.2 ± 4.8	29.6 ± 2.6	7.71 ^b^	0.001
CDR scale			-	-
CDR 0	0	130		-
CDR 0.5	60	0		-
CDR 1	38	0		-
CDR 2^+^	30	0		-

^a^ χ^2^ test and ^b^ *t*-test.

### 2.2. The mRNA Levels of Serum Calreticulin in AD Patients and Healthy Subjects

**Figure 1:**
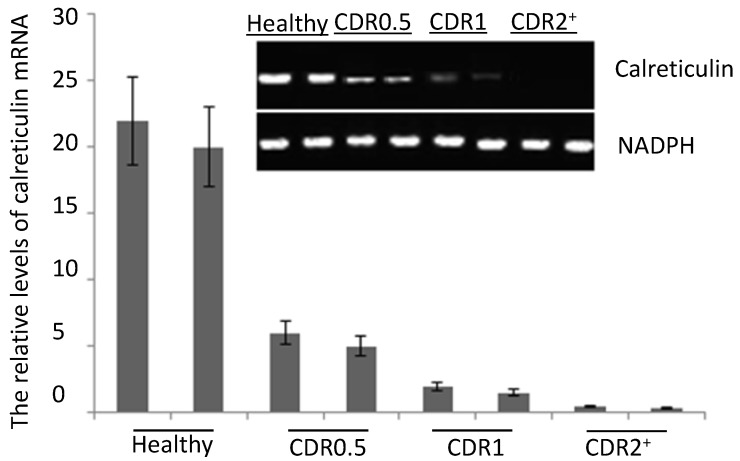
Quantitative RT-PCR analysis for the calreticulin mRNA levels in serum of Alzheimer’s disease (AD) patients and healthy participants. Quantitative RT-PCR analysis showed higher levels of calreticulin mRNA in healthy subjects than those in AD patients. Each bar represents the mean ± S.D. of three independent experiments.

The quantitative RT-PCR results showed that the mRNA levels of serum calreticulin were lower in AD patients than those from healthy subjects. The mRNA levels of serum calreticulin were decreased in the AD patients from CDR 0.5 to 2^+^. The mRNA levels of serum calreticulin were the lowest in AD patients with CDR 2^+^ (*p* < 0.05) (Figure 1). The serum mRNA levels were negatively associated with the progression of AD (*p* < 0.01).

### 2.3. ELISA Analysis

**Figure 2:**
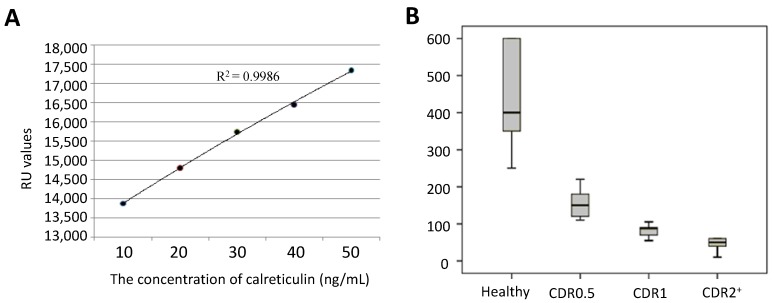
Measurement of serum calreticulin using ELISA. (**A**) A standard curve was plotted between the concentration of calreticulin and absorbing value at 450 nm and (**B**) A bar diagram shows the differences in serum levels of calreticulin among AD patients with different Clinical Dementia Rating (CDR) scores.

The concentration of calreticulin in serum was measured using a standard curve (Figure 2A). The calreticulin concentrations were 400 ± 200 ng/mL (95% CI: 350–600 ng/mL) for healthy participants, 160 ± 60 ng/mL (95% CI: 110–180 ng/mL) for AD patients with CDR 0.5, 80 ± 25 ng/mL (95% CI: 65–90 ng/mL) for AD patients with CDR 1 and 30 ± 30 ng/mL (95% CI: 20–60 ng/mL) for AD patients with CDR 2^+^ (Figure 2B). Just as for the above analyses, the serum protein levels of calreticulin were also negatively associated with the progression of AD (*p* < 0.01). 

### 2.4. Western Blot Analysis

**Figure 3:**
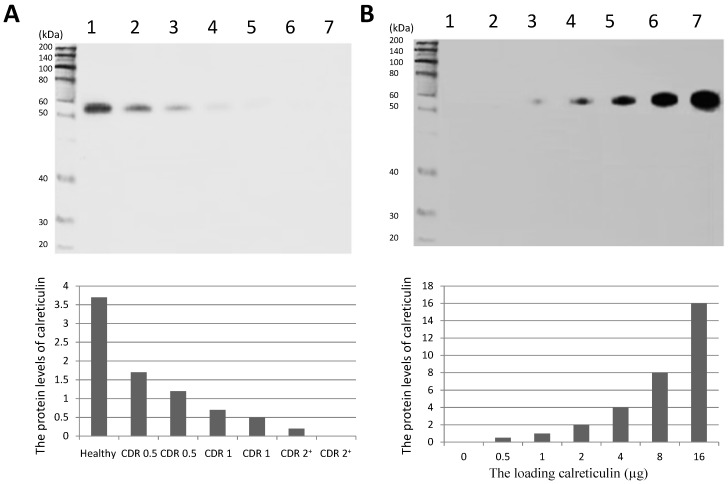
Western Blot analysis of serum calreticulin of AD patients and healthy subjects. (**A**) serum levels of calreticulin in AD patients and healthy subjects. Lane 1, a healthy control; Lanes 2–7, AD patients and (**B**) lanes 1–7, 0, 0.5, 1, 2, 4, 8 and 16 µg of calreticulin was loaded in respectively. A band of approximately 55-kDa protein could be detected (predicted molecular weight: 48 kDa).

After concentrating serum and optimizing the separating process, Western Blot analysis of calreticulin in serum was successfully performed and indicated that the density band in AD participants was lower than that from healthy subjects (Figure 3A). The linearity of the sensitivity and specificity of the calreticulin antibody were showed in Figure 3B, which suggested that the experiments were conducted well. Just as the analysis of real-time PCR and ELISA, the serum levels of calreticulin were lower in AD patients than those in a healthy group and negatively associated with the progression of AD (*p* < 0.01).

AD is a major health problem for ageing populations and is difficult to detect in an early stage. Prolonged nursing care and high cost are still needed because of the lack of a definitive therapy. AD is also a global health issue in rapidly ageing world, so it is a great challenge for dementia care. There have been more than 100 years since AD was reported in the early part of twentieth century [35]. In the past century, the technology keeping track of AD has been made a quite great progress. However, to date, the exact molecular mechanisms for degenerative changes in AD brain have not been clearly provided. AD can generally be detected after obvious changes in cognitive capacity, which leaves a little scope for reversal of these symptoms. Therefore, the normal therapeutic methods for AD remain far from satisfactory.

Ageing has been regarded as the highest risk factor for the development of AD. Progression in the prevalence of AD is doubled within every five to six years when patients are more than the age of 60, which suggests that degenerative changes in the brain is accelerating in AD patients with ageing. Thus, anti-ageing interventions can be an effective therapeutic way for the prevention of AD in the elderly population. The experiments for the anti-ageing interventions have been widely reported in animal models and calorie restriction is regarded as the most effective way for prolonging lifespan by controlling age associated illnesses [36]. A previous report finds the relationship between immunosenescence and expression of antibodies to calreticulin [37], suggesting calreticulin is associated with the anti-aging activity in mammalians.

The role of calreticulin in the prevention of brain degeneration of AD has been reported. Calreticulin is a cell surface scavenger receptor and can interact with Abeta [21] while AD pathogenesis is generally believed to be driven by the production and deposition of Abeta [38]. The composition of these amyloid betas reflects different populations of amyloid deposits, which definitely correlates with the clinical status of AD. Imaging technologies make it possible to track amyloid pathology along with disease development of AD patients. Calreticulin plays an important role for prevention of progression of AD as a cell surface scavenger receptor and whose levels in AD brains are also found to be significantly lower than those in a healthy group [20].

The major issues for detection of AD, particularly at early stages, remain a great challenge. Recently, serum biomarkers have drawn great attentions as an invasive diagnosis for AD [39,40,41,42]. However, to date, no data are available on calreticulin in the serum of AD, until the work conducted here. We found that down-regulation of serum calreticulin concentration was correlated with progression of AD according to CDR scores, which provided clinical relevance of the above observations in a Chinese population. The present study involved calreticulin was confirmed using the techniques, real-time qRT-PCR, ELISA and Western Blot. The study also demonstrated declining concentration of calreticulin in the blood of AD patients. Declined concentrations of calreticulin were most marked in AD patients and less marked in healthy subjects. Therefore, this difference of serum levels of calreticulin can be an indicator for an early diagnosis of AD.

Compared to other serum biomarkers of AD [15,16], calreticulin seems to be a more functional protein: (1) binding Ca^2+^ ions and regulating Ca^2+^ homeostasis [43]; (2) binding misfolded proteins [44]; (3) binding oligosaccharides [44]; (4) and regulation of some important signaling molecules so on [43]. The dysregulation of these functions can lead to the pathogenesis and development of AD [45,46,47,48]. For calreticulin, many new discoveries for its effects on AD will be revealed with further work. With more relevant results combining with the serum obtained by less invasive procedures, we hope that calreticulin will be widely used for the diagnosis of AD in the future. With current information, the specific role of calreticulin in AD is still unclear. However, some functions of calreticulin imply that calreticulin may be one of candidates related with the pathogenesis of AD. For example, as a Ca^2+^-sensor, calreticulin mediates Ca^2+^ homeostasis [49]. Interfering with Ca^2+^ permeability increases cerebral Abeta levels and promotes Abeta peptide deposition, which is an important risk factor for the late-onset of AD [50]. Calreticulin is a resident protein of the ER and reduced levels of calreticulin link ER stress and cell death in neurons [51], while ER stress can induce unfolded protein response and plays an important role in the pathogenesis of AD [52]. Furthermore, the neurons of AD patients are more susceptible to cell death [53] and elevated levels of calreticulin may prevent the progress of AD. The concentration of marker proteins can reflect physiological or pathological states associated with human health. We first detected the decreasing calreticulin in the serum of AD patients, which was consistent with the CDR scores. The values of serum concentrations of calreticulin were not affected by many parameters such as age and BMI.

Certainly, there are some limitations in the present work. For example, the work was not performed in a larger population, so it is necessary to conduct similar research in a larger population to increase the validity of the results. A total 258 participants were collected here including 128 AD patients and 130 healthy participants. The sample size seems a little small for the study of serum calreticulin. The main difficulty was caused by the fact that it was difficult to recruit more AD patients according to inclusion and exclusion criteria. On the other hand, many AD patients cast doubts on present techniques because AD could not be cured in most cases. Furthermore, a biomarker did not present a complete story of AD causality. In any case, the present work will be useful for better understanding the molecular mechanism for causing AD.

## 3. Experimental Section

### 3.1. Participants

All experiments were approved by the ethics committee from the Fourth Affiliated Hospital of China Medical University (Shenyang, China). A written informed consent was signed by all randomly recruited participants. From May 2010 to July 2013 a total of 128 patients were diagnosed for AD. AD patients were confirmed by screening cognitive impairment with Mini Mental State Examination (MMSE scores ≤ 24) and a neuro-psychological test via Clinical Dementia Rating (CDR). All AD patients had an overall CDR of 0.5 or over 0.5. AD patients were diagnosed according to NINCDS-ADRDA criteria [54]. To further diagnose AD based on imaging-based techniques, the magnetic resonance image (MRI) relaxation time constant was examined in the brains of AD patients according to a previous report [55]. Neuropsychological impairment was measured according to Global Deficit Score (GDS) for classifying Neuropsychological impairment [56]. A total of 130 elderly healthy individuals (MMSE scores > 28, CDR = 0) were randomly recruited as controls. All healthy subjects had no brain diseases with the assessment of the MRI and CT. Meanwhile, they had no history or symptoms of ischemic or hemorrhagic stroke.

All the persons including AD patients and healthy participants were unrelated ethnic Han Chinese. All the subjects shared a similar cultural and economic background. All subjects were randomly recruited from the same place with healthy livers and kidneys. Other parameters, such as blood pressure, pulmonary function, electrocardiography and chest radiography were in fine results in all subjects. AD is affected by age, gender, education level, BMI (body mass index) and life habits (smoking and drinking) so on. Thus, all these parameters were constrained to keep no heterogeneous results between AD patients and healthy subjects.

### 3.2. mRNA Extraction

Five milliliters of venous blood was taken from each individual. The serum was collected after centrifugation of venous blood at 3000× *g* for 10 min. Total RNA was extracted from 1 mL serum using the serum RNA extraction kit (2BScientific Ltd., Upper Heyford, UK).

### 3.3. Real-Time qRT-PCR

One microliter of mRNA was reverse-transcribed into cDNA in a 10 μL total volume using AMV reverse transcriptase (TaKaRa Biotechnology (Dalian) Co., Ltd., Dalian, China). Five microliter from the reverse-transcribed mixture were amplified by real-time qPCR in a 20 μL volume with primers for calreticulin (Forward primer, 5'-aggatgatgagtttacacac-3'; Reverse primer, 5'-tcatcgatcttggcccgctc-3') and GAPDH (Forward primer, 5'-tcaagatcatcagcaatgcc-3'; Reverse primer, 5'-gaccttgcccacagccttgg-3'). PCR was performed using LightCycler^®^ FastStart DNA Master SYBR Green I with qTOWER 2.0 (Analytik Jena, Jena, Germany). The PCR condition was designed as 40 cycles of denaturing (95 °C for 5 s), annealing (60 °C for 5 s) and extension (72 °C for 15 s). To control the integrity between the sample and inter-sample in mRNA level, GAPDH gene was amplified under the same condition.

### 3.4. ELISA

Microtiter plates were coated with serum sample in each well with the same volume and ELISA was performed by using ELISA Kit for calreticulin (CRT) according to an instruction manual (SEB486Hu, Uscn Life Science Inc., Wuhan, China). The absorption value for Nitrophenolate 158 was measured at 405 nm using Automated ELISA analyzer (Yantai Addcare Bio-Tech Co., Ltd., Yantai, China). The series of different concentrations of calreticulin were used to plot a standard curve.

### 3.5. Western Blot

To confirm the existence of calreticulin in serum, serum samples from AD patients and controls were prepared in high quality via the removal of interfering proteins using Multiple Affinity Removal Spin Cartridge (Agilent Technologies, Santa Clara, CA, USA). The fluid was concentrated using centricon column with a 30-kDa cutoff value (Millipore, Billerica, MA, USA). The concentration of total proteins was determined using BCA protein quantification kit (Pierce, Rockford, IL, USA). Western Blot was conducted using Calreticulin Antibody (1:1000) (MA5-15382, Thermo Fisher Scientific Inc., Rockford, IL, USA) and goat anti-mouse IgG-HRP (sc-2005, Santa Cruz Biotechnology, Santa Cruz, CA, USA). Immunoreactive bands were measured by ECL (GE Healthcare, Beijing, China) and densitometry was quantified using Image J software 1.45 (NIH, Bethesda, MD, USA).

### 3.6. Statistical Analysis

All the variables in serum between AD patients and healthy subjects were compared using a *t*-test. *p* < 0.05 was considered statistically significant.

## 4. Conclusions

We find a decrease of serum levels of calreticulin in AD patients, which can predict the progression of AD in patients. Serum levels of calreticulin can be a negative biomarker for early diagnosis of AD. Further research to determine the molecular mechanisms whether calreticulin prevents the development of AD in a larger population is much needed. Based on all the work, an effective therapeutic method can be developed for the therapy of AD.
